# Butane-1,4-diammonium bis­(pyridine-2,6-dicarboxyl­ato)cuprate(II) trihydrate

**DOI:** 10.1107/S1600536808011938

**Published:** 2008-04-30

**Authors:** Hossein Aghabozorg, Najmeh Firoozi, Leila Roshan, Jafar Attar Gharamaleki, Mohammad Ghadermazi

**Affiliations:** a Faculty of Chemistry, Tarbiat Moallem University, 49 Mofateh Avenue, Tehran, Iran; b Department of Chemistry, Faculty of Science, University of Kurdistan, Sanandaj, Iran

## Abstract

In the title compound, (C_4_H_14_N_2_)[Cu(C_7_H_3_NO_4_)_2_]·3H_2_O or (bdaH_2_)[Cu(pydc)_2_]·3H_2_O (where bda is butane-1,4-diamine and pydcH_2_ is pyridine-2,6-dicarboxylic acid), the Cu^II^ atom is coordinated by four O atoms [Cu—O = 2.0557 (16)–2.3194 (16) Å] and two N atoms [Cu—N = 1.9185 (18) and 1.9638 (18) Å] from two chelating rings of the pydc^2−^ anions, which act as tridentate ligands. The geometry of the resulting CuN_2_O_4_ coordination can be described as distorted octa­hedral. The the two pydc^2−^ fragments are almost perpendicular to one another [77.51 (11)°]. To balance the charges, two centrosymmetric protonated butane-1,4-diammonium, (bdaH_2_)^2+^ cations are present. In the crystal structure, extensive O—H⋯O, N—H⋯O and C—H⋯O hydrogen bonds [*D*⋯*A* = 2.720 (2)–3.446 (3) Å], ion pairing, C—O⋯π [O⋯π = 3.099 (2) Å] and π–π stacking inter­actions between the pydc^2−^ rings [centroid–centroid distance = 3.5334 (15) Å] contribute to the formation of a three-dimensional supra­molecular structure.

## Related literature

For related literature, see: Aghabozorg *et al.* (2006 ▶, 2008*a*,*b*,*c* ▶).
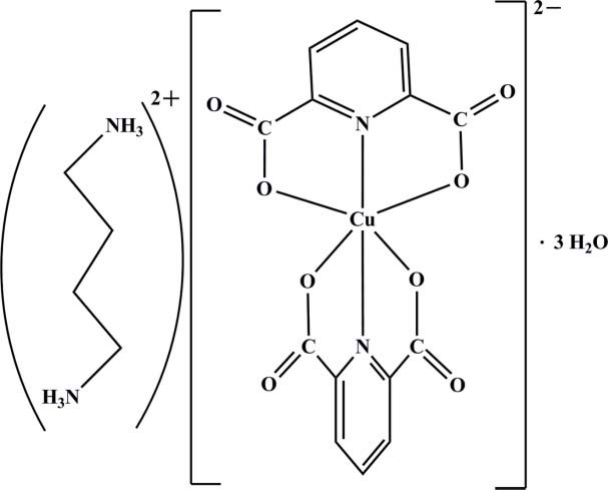


## Experimental

### 

#### Crystal data

(C_4_H_14_N_2_)[Cu(C_7_H_3_NO_4_)_2_]·3H_2_O
*M* *_r_* = 537.97
Triclinic, 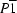

*a* = 8.0931 (13) Å
*b* = 11.4017 (19) Å
*c* = 12.977 (2) Å
α = 71.632 (5)°
β = 89.195 (5)°
γ = 72.892 (5)°
*V* = 1082.1 (3) Å^3^
*Z* = 2
Mo *K*α radiation
μ = 1.08 mm^−1^
*T* = 100 (2) K
0.25 × 0.20 × 0.20 mm

#### Data collection

Bruker SMART APEXII CCD area-detector diffractometer
Absorption correction: multi-scan (*APEX2*; Bruker, 2005 ▶) *T*_min_ = 0.775, *T*_max_ = 0.815
10991 measured reflections
5185 independent reflections
4097 reflections with *I* > 2/s(*I*)
*R*_int_ = 0.035

#### Refinement

*R*[*F* ^2^ > 2σ(*F* ^2^)] = 0.037
*wR*(*F* ^2^) = 0.092
*S* = 1.01
5185 reflections
307 parameters
H-atom parameters constrained
Δρ_max_ = 0.43 e Å^−3^
Δρ_min_ = −0.50 e Å^−3^

### 

Data collection: *APEX2* (Bruker, 2005 ▶); cell refinement: *APEX2*; data reduction: *APEX2*; program(s) used to solve structure: *SHELXTL* (Sheldrick, 2008 ▶); program(s) used to refine structure: *SHELXTL*; molecular graphics: *SHELXTL*; software used to prepare material for publication: *SHELXTL*.

## Supplementary Material

Crystal structure: contains datablocks I, global. DOI: 10.1107/S1600536808011938/su2053sup1.cif

Structure factors: contains datablocks I. DOI: 10.1107/S1600536808011938/su2053Isup2.hkl

Additional supplementary materials:  crystallographic information; 3D view; checkCIF report

## Figures and Tables

**Table 1:** Hydrogen-bond geometry (Å, °)

*D*—H⋯*A*	*D*—H	H⋯*A*	*D*⋯*A*	*D*—H⋯*A*
O1*W*—H1*WA*⋯O3^i^	0.85	1.91	2.725 (2)	160
O1*W*—H1*WB*⋯O6^ii^	0.85	1.89	2.720 (2)	167
O2*W*—H2*WA*⋯O1*W*^iii^	0.85	1.94	2.771 (2)	165
O2*W*—H2*WB*⋯O2	0.85	1.98	2.828 (2)	174
O3*W*—H3*WA*⋯O1*W*^iii^	0.85	2.03	2.874 (3)	171
O3*W*—H3*WB*⋯O2	0.85	1.97	2.779 (3)	158
N1*S*—H1*NA*⋯O4	0.91	1.90	2.804 (3)	171
N1*S*—H1*NB*⋯O7^iv^	0.83	2.55	3.112 (3)	126
N1*S*—H1*NB*⋯O8^iv^	0.83	2.04	2.865 (2)	176
N1*S*—H1*NC*⋯O2*W*^v^	0.84	2.28	2.895 (3)	131
N1*S*—H1*NC*⋯O4^vi^	0.84	2.28	2.981 (3)	141
N2*S*—H2*NA*⋯O3*W*^iv^	0.79	1.95	2.730 (3)	166
N2*S*—H2*NB*⋯O5	0.86	2.31	3.149 (3)	164
N2*S*—H2*NB*⋯O6	0.86	2.31	3.001 (3)	138
N2*S*—H2*NC*⋯O2*W*	0.87	2.00	2.867 (3)	173
C10—H10*A*⋯O3^vii^	0.95	2.58	3.446 (3)	151
C11—H11*A*⋯O1^viii^	0.95	2.46	3.139 (3)	128
C3*S*—H3*SA*⋯O8^ix^	0.99	2.54	3.178 (3)	122

Symmetry codes: (i) 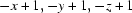; (ii) 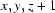; (iii) 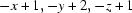; (iv) 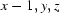; (v) 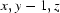; (vi) 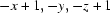; (vii) 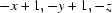; (viii) 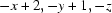; (ix) 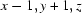.

**Fig. 1.**
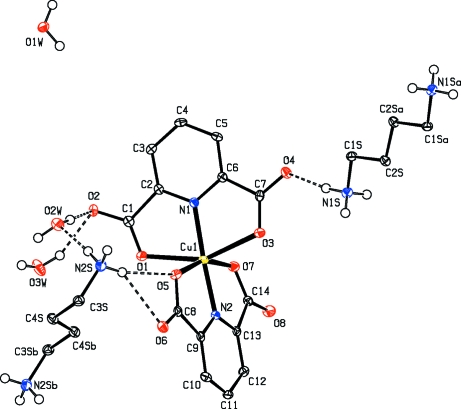
The molecular structure of the title compound, with displacement ellipsoids drawn at the 50% probability level. Atoms marked with a and b are related by the symmetry codes (-x, -y, -z + 1) and (-x + 1, -y + 2, -z), respectively. Hydrogen bonds are shown as dashedlines. Hydrogen atoms are not involved in the hydrogen bonding are omitted for clarity.

**Fig. 2.**
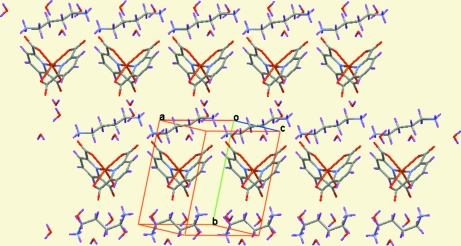
A layered packing diagram of the title compound. The space between the two layers of [Cu(pydc)2]2– anions is filled with a layer of (bdaH2)2+ cations and water molecules.

**Fig. 3.**
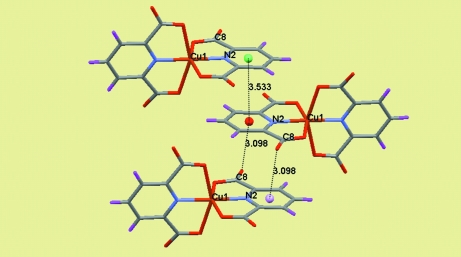
A view of the π-π stacking interactions, between the aromatic rings of the pydc2- dianions with distances of 3.5334 (15) for Cg1···Cg1 [2-x, 1 - y, -z], and the C—O···π stacking interactions, between the carbonyl groups of the pyridine-2,6-dicarboxylate groups and the pydc2- fragments: distance O···π is 3.099 (2) Å for C8—O6···Cg1 (1 - x, 1 - y, -z) [Cg1 is the centroid for ring (N2,C9—C13)].

**Fig. 4.**
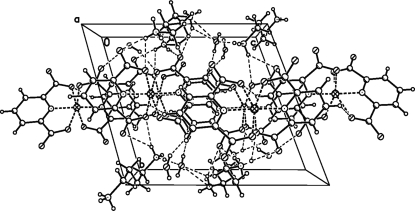
The crystal packing of the title compound, viewed along the a axis, with the hydrogen bonds shown as dashed lines.

## supplementary crystallographic information

### Comment

In order to study the hydrogen-bonding patterns in proton-transfer compounds, our research group has selected pyridine-2,6-dicarboxylic acid (pydcH_2_) and 1,10-phenanthroline-2,9-dicarboxylic acid (phendcH_2_) as proton donors, and piperazine (pipz), creatinine (creat) and 1,10-phenanthroline (phen) as proton acceptors. This has resulted in the formation of new proton-transfer systems, such as (pipzH_2_)(pydc) (Aghabozorg *et al.*, 2006). In this regard, we have so far synthesized several metal organic complexes (Aghabozorg, *et al.*, 2008*a*; 2008*b*, 2008*c*).

The molecular structure of the title compound is shown in Fig. 1. Hydrogen bond geometries are given in Table 1. The Cu^II^ atom is six-coordinated by two pyridine-2,6-dicarboxylate, or pydc^2-^ anions; *i.e.* each pydc^2-^ anion is coordinated through one pyridine N atom and two carboxylate O atoms. Atoms N1 and N2 of two pydc^2-^ fragments occupy the axial positions, while atoms O1, O3, O5 and O7 form the equatorial plane. The N1–Cu1–N2 angle [177.14 (8)^°^] deviates slightly from linearity. Therefore, the geometry of the resulting CuN_2_O_4_ coordination can be described as distorted octahedral. The Cu1–O1 and Cu1–O3 bond distances [2.2824 (17) and 2.3194 (16) Å, respectively] are longer than the other metal-ligand bonds, perhaps due to the pseudo Jahn-Teller effect. The bond angles O1–Cu1–O5 and O3–Cu1–O7 are 87.02 (6)^°^ and 89.89 (6)^°^, respectively, and the O5–Cu1–O1–C1 and O7–Cu1–O3–C7 torsion angles are 90.51 (15)^°^and 93.33 (15)^°^, respectively. The angle between the two mean planes passing through the pydc^2-^ cations is 77.51 (11)^°^, indicating that these two units are almost perpendicular to one another. Furthermore, the bond angles O1–Cu1–O3 [153.33 (6)^°^] and O5–Cu1–O7 [159.68 (6)^°^] indicate that the four carboxylate groups of the two dianions are oriented in a flattened tetrahedral arrangement around the Cu^II^ atom.

In the crystal structure of the title complex there are three water molecules of crystallization, and two centrosymmetric butane-1,4-diammonium cations present as counter-ions. The spaces between two layers of [Cu(pydc)_2_]^2–^ dianions are filled with (bnH_2_)^2+^ cations and water molecules (Fig. 2). There are also π-π stacking interactions between the aromatic rings of the coordinated pydc^2-^ dianions, with distances of 3.5334 (15) Å for *Cg*1···*Cg*1 [2*-x*, 1 - *y*, *-z*]. There are also C–O···π stacking interactions between the carbonyl groups of the pyridine-2,6-dicarboxylate groups and the pyridine ring of symmetry related dications, with an O···π distance of 3.099 (2) Å (measured to the center of the pyridine ring) for C8–O6···*Cg*1 (1 - *x*, 1 - *y*, -*z*) [*Cg*1 is the centroid for the (N2,C9—C13) ring] (see Fig. 3).

In the crystal structure there are O–H···O, N–H···O and C–H···O hydrogen bonds, with D···A distances ranging from 2.720 (2) to 3.446 (3) Å, which result in the formation of a supramolecular structure (Fig. 4). Ion pairing, π–π and C–O···π stacking interactions are also effective in the crystal packing.

### Experimental

A mixture of an aqueous solution (30 ml) of the proton transfer compound (bdaH_2_)(pydc) (100 mg, 0.4 mmol) and copper(II) chloride dihydrate (30 mg, 0.2 mmol) were stirred at room temperature. Blue crystals of the title compound were obtained after four weeks at room temperature.

### Refinement

The hydrogen atoms of the water molecules and the NH groups were located in difference Fourier syntheses. The C-bound H-atoms were included in calculated positions. All the hydrogen atoms were treated as riding atoms: O—H = 0.85, N—H = 0.79 - 0.91, C—H = 0.95 - 0.99 Å with *U*_iso_(H) = 1.2 or 1.5*U*_eq_(parent O, N or C atom).

### Crystal data

**Table:**

(C_4_H_14_N_2_)[Cu(C_7_H_3_NO_4_)_2_]·3H_2_O	*Z* = 2
*M**_r_* = 537.97	*F*_000_ = 558
Triclinic, *P*1	*D*_x_ = 1.651 Mg m^−^^3^
Hall symbol: -P 1	Mo *K*α radiation λ = 0.71073 Å
*a* = 8.0931 (13) Å	Cell parameters from 657 reflections
*b* = 11.4017 (19) Å	θ = 3–30º
*c* = 12.977 (2) Å	µ = 1.08 mm^−^^1^
α = 71.632 (5)º	*T* = 100 (2) K
β = 89.195 (5)º	Prism, blue
γ = 72.892 (5)º	0.25 × 0.20 × 0.20 mm
*V* = 1082.1 (3) Å^3^	

### Data collection

**Table:**

Bruker SMART APEXII CCD area-detector diffractometer	5185 independent reflections
Radiation source: fine-focus sealed tube	4097 reflections with *I* > 2/s(*I*)
Monochromator: graphite	*R*_int_ = 0.035
*T* = 100(2) K	θ_max_ = 28.0º
φ and ω scans	θ_min_ = 1.7º
Absorption correction: multi-scan(APEX2; Bruker, 2005)	*h* = −10→10
*T*_min_ = 0.775, *T*_max_ = 0.815	*k* = −15→15
10991 measured reflections	*l* = −17→17

### Refinement

**Table:**

Refinement on *F*^2^	Secondary atom site location: difference Fourier map
Least-squares matrix: full	Hydrogen site location: inferred from neighbouring sites
*R*[*F*^2^ > 2σ(*F*^2^)] = 0.037	H-atom parameters constrained
*wR*(*F*^2^) = 0.092	*w* = 1/[σ^2^(*F*_o_^2^) + (0.046*P*)^2^ + 0.23*P*] where *P* = (*F*_o_^2^ + 2*F*_c_^2^)/3
*S* = 1.01	(Δ/σ)_max_ = 0.001
5185 reflections	Δρ_max_ = 0.43 e Å^−^^3^
307 parameters	Δρ_min_ = −0.50 e Å^−^^3^
Primary atom site location: structure-invariant direct methods	Extinction correction: none

### Special details

**Table:**

Geometry. All e.s.d.'s (except the e.s.d. in the dihedral angle between two l.s. planes) are estimated using the full covariance matrix. The cell e.s.d.'s are taken into account individually in the estimation of e.s.d.'s in distances, angles and torsion angles; correlations between e.s.d.'s in cell parameters are only used when they are defined by crystal symmetry. An approximate (isotropic) treatment of cell e.s.d.'s is used for estimating e.s.d.'s involving l.s. planes.
Refinement. Refinement of *F*^2^ against ALL reflections. The weighted *R*-factor *wR* and goodness of fit *S* are based on *F*^2^, conventional *R*-factors *R* are based on *F*, with *F* set to zero for negative *F*^2^. The threshold expression of *F*^2^ > σ(*F*^2^) is used only for calculating *R*-factors(gt) *etc*. and is not relevant to the choice of reflections for refinement. *R*-factors based on *F*^2^ are statistically about twice as large as those based on *F*, and *R*- factors based on ALL data will be even larger.

### Fractional atomic coordinates and isotropic or equivalent isotropic displacement parameters (Å^2^)

**Table:**

	*x*	*y*	*z*	*U*_iso_*/*U*_eq_	
Cu1	0.75989 (4)	0.45400 (3)	0.24669 (2)	0.00985 (9)	
N1	0.7337 (2)	0.44458 (18)	0.39951 (15)	0.0101 (4)	
N2	0.7825 (2)	0.45531 (18)	0.09900 (14)	0.0100 (4)	
O1	0.8798 (2)	0.60585 (16)	0.26295 (13)	0.0140 (3)	
O2	0.8552 (2)	0.72368 (15)	0.37585 (13)	0.0132 (3)	
O3	0.6132 (2)	0.30200 (16)	0.31081 (12)	0.0149 (3)	
O4	0.5355 (2)	0.19437 (15)	0.47134 (13)	0.0140 (3)	
O5	0.5403 (2)	0.60836 (15)	0.16832 (12)	0.0131 (3)	
O6	0.4101 (2)	0.72403 (15)	0.00191 (13)	0.0140 (3)	
O7	0.9888 (2)	0.30660 (15)	0.26742 (12)	0.0138 (3)	
O8	1.1597 (2)	0.18855 (16)	0.17599 (13)	0.0160 (4)	
C1	0.8456 (3)	0.6277 (2)	0.35079 (18)	0.0107 (4)	
C2	0.7821 (3)	0.5280 (2)	0.43635 (17)	0.0095 (4)	
C3	0.7693 (3)	0.5226 (2)	0.54446 (18)	0.0114 (4)	
H3A	0.8029	0.5825	0.5699	0.014*	
C4	0.7071 (3)	0.4291 (2)	0.61469 (18)	0.0125 (5)	
H4A	0.7002	0.4226	0.6893	0.015*	
C5	0.6547 (3)	0.3445 (2)	0.57476 (17)	0.0115 (4)	
H5A	0.6108	0.2799	0.6215	0.014*	
C6	0.6676 (3)	0.3563 (2)	0.46554 (17)	0.0098 (4)	
C7	0.6003 (3)	0.2760 (2)	0.41193 (18)	0.0106 (4)	
C8	0.5269 (3)	0.6347 (2)	0.06519 (18)	0.0109 (4)	
C9	0.6692 (3)	0.5474 (2)	0.02072 (18)	0.0104 (4)	
C10	0.6882 (3)	0.5581 (2)	−0.08775 (18)	0.0118 (4)	
H10A	0.6083	0.6244	−0.1441	0.014*	
C11	0.8295 (3)	0.4675 (2)	−0.11138 (18)	0.0113 (4)	
H11A	0.8469	0.4723	−0.1850	0.014*	
C12	0.9442 (3)	0.3708 (2)	−0.02782 (18)	0.0119 (4)	
H12A	1.0393	0.3083	−0.0432	0.014*	
C13	0.9168 (3)	0.3674 (2)	0.07877 (18)	0.0101 (4)	
C14	1.0324 (3)	0.2783 (2)	0.18158 (18)	0.0111 (4)	
O1W	0.3016 (2)	0.87914 (15)	0.79263 (13)	0.0169 (4)	
H1WA	0.3265	0.8381	0.7472	0.020*	
H1WB	0.3197	0.8298	0.8586	0.020*	
O2W	0.5445 (2)	0.92612 (16)	0.28473 (13)	0.0167 (4)	
H2WA	0.5891	0.9844	0.2501	0.020*	
H2WB	0.6336	0.8614	0.3129	0.020*	
O3W	0.9924 (2)	0.89746 (17)	0.22672 (15)	0.0242 (4)	
H3WA	0.9134	0.9687	0.2188	0.029*	
H3WB	0.9543	0.8346	0.2593	0.029*	
N1S	0.3148 (2)	0.08444 (18)	0.39577 (15)	0.0125 (4)	
H1NA	0.3827	0.1273	0.4137	0.015*	
H1NB	0.2687	0.1187	0.3324	0.015*	
H1NC	0.3800	0.0095	0.4038	0.015*	
C1S	0.1856 (3)	0.0767 (2)	0.47831 (18)	0.0120 (4)	
H1SA	0.1216	0.1652	0.4785	0.014*	
H1SB	0.2466	0.0264	0.5516	0.014*	
C2S	0.0584 (3)	0.0125 (2)	0.45404 (18)	0.0119 (4)	
H2SA	0.1232	−0.0708	0.4437	0.014*	
H2SB	−0.0137	0.0690	0.3855	0.014*	
N2S	0.3056 (3)	0.89178 (19)	0.14406 (16)	0.0151 (4)	
H2NA	0.2235	0.8823	0.1757	0.018*	
H2NB	0.3607	0.8177	0.1379	0.018*	
H2NC	0.3714	0.9017	0.1909	0.018*	
C3S	0.2648 (3)	0.9975 (2)	0.03651 (18)	0.0139 (5)	
H3SA	0.1623	1.0688	0.0405	0.017*	
H3SB	0.2351	0.9636	−0.0199	0.017*	
C4S	0.4156 (3)	1.0503 (2)	0.00368 (19)	0.0146 (5)	
H4SA	0.4374	1.0909	0.0571	0.017*	
H4SB	0.3820	1.1193	−0.0681	0.017*	

### Atomic displacement parameters (Å^2^)

**Table:**

	*U*^11^	*U*^22^	*U*^33^	*U*^12^	*U*^13^	*U*^23^
Cu1	0.01106 (15)	0.01085 (14)	0.00716 (13)	−0.00274 (10)	0.00140 (10)	−0.00287 (10)
N1	0.0097 (9)	0.0090 (9)	0.0113 (9)	−0.0023 (7)	0.0021 (7)	−0.0034 (7)
N2	0.0120 (10)	0.0099 (9)	0.0085 (9)	−0.0045 (8)	0.0018 (7)	−0.0028 (7)
O1	0.0172 (9)	0.0154 (8)	0.0119 (8)	−0.0077 (7)	0.0050 (6)	−0.0056 (7)
O2	0.0139 (8)	0.0107 (8)	0.0163 (8)	−0.0051 (6)	0.0013 (6)	−0.0050 (7)
O3	0.0213 (9)	0.0158 (8)	0.0099 (8)	−0.0087 (7)	0.0021 (6)	−0.0047 (7)
O4	0.0144 (8)	0.0129 (8)	0.0143 (8)	−0.0063 (7)	0.0013 (6)	−0.0022 (7)
O5	0.0133 (8)	0.0150 (8)	0.0113 (8)	−0.0035 (7)	0.0014 (6)	−0.0056 (7)
O6	0.0142 (8)	0.0113 (8)	0.0141 (8)	−0.0027 (7)	0.0003 (6)	−0.0018 (6)
O7	0.0157 (9)	0.0129 (8)	0.0100 (8)	−0.0017 (7)	−0.0003 (6)	−0.0022 (6)
O8	0.0146 (9)	0.0150 (8)	0.0145 (8)	0.0008 (7)	−0.0002 (7)	−0.0044 (7)
C1	0.0063 (10)	0.0110 (11)	0.0126 (10)	−0.0008 (8)	−0.0014 (8)	−0.0023 (9)
C2	0.0058 (10)	0.0096 (10)	0.0117 (10)	−0.0017 (8)	0.0005 (8)	−0.0023 (8)
C3	0.0101 (11)	0.0128 (11)	0.0120 (10)	−0.0025 (9)	0.0004 (8)	−0.0059 (9)
C4	0.0114 (11)	0.0166 (12)	0.0078 (10)	−0.0018 (9)	−0.0002 (8)	−0.0040 (9)
C5	0.0112 (11)	0.0129 (11)	0.0080 (10)	−0.0036 (9)	0.0004 (8)	−0.0004 (9)
C6	0.0076 (10)	0.0084 (10)	0.0111 (10)	−0.0002 (8)	0.0007 (8)	−0.0024 (8)
C7	0.0088 (11)	0.0077 (10)	0.0138 (11)	−0.0004 (8)	−0.0007 (8)	−0.0036 (9)
C8	0.0114 (11)	0.0105 (11)	0.0134 (10)	−0.0068 (9)	0.0024 (8)	−0.0044 (9)
C9	0.0113 (11)	0.0088 (10)	0.0122 (10)	−0.0055 (9)	0.0011 (8)	−0.0027 (9)
C10	0.0106 (11)	0.0149 (11)	0.0097 (10)	−0.0063 (9)	−0.0002 (8)	−0.0016 (9)
C11	0.0133 (11)	0.0147 (11)	0.0087 (10)	−0.0079 (9)	0.0021 (8)	−0.0041 (9)
C12	0.0106 (11)	0.0147 (11)	0.0136 (11)	−0.0062 (9)	0.0023 (9)	−0.0068 (9)
C13	0.0111 (11)	0.0093 (10)	0.0120 (10)	−0.0057 (9)	0.0033 (8)	−0.0041 (9)
C14	0.0118 (11)	0.0109 (11)	0.0108 (10)	−0.0049 (9)	0.0020 (8)	−0.0026 (9)
O1W	0.0249 (10)	0.0128 (8)	0.0112 (8)	−0.0028 (7)	0.0002 (7)	−0.0044 (7)
O2W	0.0137 (8)	0.0155 (9)	0.0175 (8)	−0.0026 (7)	0.0012 (7)	−0.0027 (7)
O3W	0.0194 (10)	0.0143 (9)	0.0338 (11)	−0.0049 (7)	0.0123 (8)	−0.0016 (8)
N1S	0.0115 (10)	0.0126 (10)	0.0122 (9)	−0.0017 (8)	−0.0018 (7)	−0.0042 (8)
C1S	0.0128 (11)	0.0138 (11)	0.0101 (10)	−0.0047 (9)	0.0025 (8)	−0.0044 (9)
C2S	0.0113 (11)	0.0116 (11)	0.0121 (11)	−0.0044 (9)	0.0003 (9)	−0.0022 (9)
N2S	0.0150 (10)	0.0154 (10)	0.0182 (10)	−0.0070 (8)	0.0073 (8)	−0.0079 (8)
C3S	0.0135 (11)	0.0163 (12)	0.0135 (11)	−0.0056 (9)	0.0025 (9)	−0.0060 (9)
C4S	0.0154 (12)	0.0142 (12)	0.0145 (11)	−0.0064 (10)	0.0036 (9)	−0.0036 (9)

### Geometric parameters (Å, °)

**Table:**

Cu1—N2	1.9185 (18)	C11—C12	1.388 (3)
Cu1—N1	1.9638 (18)	C11—H11A	0.9500
Cu1—O7	2.0557 (16)	C12—C13	1.388 (3)
Cu1—O5	2.0909 (16)	C12—H12A	0.9500
Cu1—O1	2.2824 (17)	C13—C14	1.519 (3)
Cu1—O3	2.3194 (16)	O1W—H1WA	0.8500
N1—C6	1.339 (3)	O1W—H1WB	0.8500
N1—C2	1.341 (3)	O2W—H2WA	0.8500
N2—C9	1.328 (3)	O2W—H2WB	0.8500
N2—C13	1.331 (3)	O3W—H3WA	0.8500
O1—C1	1.252 (3)	O3W—H3WB	0.8499
O2—C1	1.259 (3)	N1S—C1S	1.487 (3)
O3—C7	1.261 (3)	N1S—H1NA	0.9103
O4—C7	1.249 (3)	N1S—H1NB	0.8297
O5—C8	1.275 (3)	N1S—H1NC	0.8359
O6—C8	1.238 (3)	C1S—C2S	1.514 (3)
O7—C14	1.272 (3)	C1S—H1SA	0.9900
O8—C14	1.240 (3)	C1S—H1SB	0.9900
C1—C2	1.524 (3)	C2S—C2S^i^	1.520 (4)
C2—C3	1.389 (3)	C2S—H2SA	0.9900
C3—C4	1.383 (3)	C2S—H2SB	0.9900
C3—H3A	0.9500	N2S—C3S	1.493 (3)
C4—C5	1.393 (3)	N2S—H2NA	0.7927
C4—H4A	0.9500	N2S—H2NB	0.8595
C5—C6	1.386 (3)	N2S—H2NC	0.8687
C5—H5A	0.9500	C3S—C4S	1.513 (3)
C6—C7	1.525 (3)	C3S—H3SA	0.9900
C8—C9	1.523 (3)	C3S—H3SB	0.9900
C9—C10	1.385 (3)	C4S—C4S^ii^	1.529 (5)
C10—C11	1.401 (3)	C4S—H4SA	0.9900
C10—H10A	0.9500	C4S—H4SB	0.9900
			
N2—Cu1—N1	177.14 (8)	C9—C10—H10A	121.2
N2—Cu1—O7	80.03 (7)	C11—C10—H10A	121.2
N1—Cu1—O7	99.13 (7)	C12—C11—C10	120.3 (2)
N2—Cu1—O5	79.73 (7)	C12—C11—H11A	119.8
N1—Cu1—O5	101.17 (7)	C10—C11—H11A	119.8
O7—Cu1—O5	159.68 (6)	C11—C12—C13	118.5 (2)
N2—Cu1—O1	105.55 (7)	C11—C12—H12A	120.8
N1—Cu1—O1	77.25 (7)	C13—C12—H12A	120.8
O7—Cu1—O1	96.69 (6)	N2—C13—C12	120.0 (2)
O5—Cu1—O1	87.02 (6)	N2—C13—C14	112.18 (19)
N2—Cu1—O3	101.03 (7)	C12—C13—C14	127.6 (2)
N1—Cu1—O3	76.20 (7)	O8—C14—O7	125.7 (2)
O7—Cu1—O3	89.89 (6)	O8—C14—C13	119.51 (19)
O5—Cu1—O3	95.71 (6)	O7—C14—C13	114.77 (19)
O1—Cu1—O3	153.33 (6)	H1WA—O1W—H1WB	113.4
C6—N1—C2	120.69 (19)	H2WA—O2W—H2WB	102.3
C6—N1—Cu1	120.25 (15)	H3WA—O3W—H3WB	109.4
C2—N1—Cu1	119.06 (15)	C1S—N1S—H1NA	105.8
C9—N2—C13	122.70 (19)	C1S—N1S—H1NB	112.6
C9—N2—Cu1	118.88 (15)	H1NA—N1S—H1NB	113.5
C13—N2—Cu1	118.33 (15)	C1S—N1S—H1NC	108.8
C1—O1—Cu1	110.18 (14)	H1NA—N1S—H1NC	106.0
C7—O3—Cu1	111.42 (14)	H1NB—N1S—H1NC	109.8
C8—O5—Cu1	113.58 (14)	N1S—C1S—C2S	110.98 (18)
C14—O7—Cu1	114.42 (14)	N1S—C1S—H1SA	109.4
O1—C1—O2	127.4 (2)	C2S—C1S—H1SA	109.4
O1—C1—C2	116.34 (19)	N1S—C1S—H1SB	109.4
O2—C1—C2	116.26 (19)	C2S—C1S—H1SB	109.4
N1—C2—C3	120.8 (2)	H1SA—C1S—H1SB	108.0
N1—C2—C1	114.97 (19)	C1S—C2S—C2S^i^	111.3 (2)
C3—C2—C1	124.2 (2)	C1S—C2S—H2SA	109.4
C4—C3—C2	119.2 (2)	C2S^i^—C2S—H2SA	109.4
C4—C3—H3A	120.4	C1S—C2S—H2SB	109.4
C2—C3—H3A	120.4	C2S^i^—C2S—H2SB	109.4
C3—C4—C5	119.3 (2)	H2SA—C2S—H2SB	108.0
C3—C4—H4A	120.4	C3S—N2S—H2NA	114.7
C5—C4—H4A	120.4	C3S—N2S—H2NB	112.1
C6—C5—C4	118.8 (2)	H2NA—N2S—H2NB	106.2
C6—C5—H5A	120.6	C3S—N2S—H2NC	114.7
C4—C5—H5A	120.6	H2NA—N2S—H2NC	103.8
N1—C6—C5	121.1 (2)	H2NB—N2S—H2NC	104.4
N1—C6—C7	115.97 (19)	N2S—C3S—C4S	111.92 (19)
C5—C6—C7	122.8 (2)	N2S—C3S—H3SA	109.2
O4—C7—O3	127.0 (2)	C4S—C3S—H3SA	109.2
O4—C7—C6	117.28 (19)	N2S—C3S—H3SB	109.2
O3—C7—C6	115.65 (19)	C4S—C3S—H3SB	109.2
O6—C8—O5	124.9 (2)	H3SA—C3S—H3SB	107.9
O6—C8—C9	119.90 (19)	C3S—C4S—C4S^ii^	114.9 (2)
O5—C8—C9	115.19 (19)	C3S—C4S—H4SA	108.5
N2—C9—C10	120.8 (2)	C4S^ii^—C4S—H4SA	108.5
N2—C9—C8	112.45 (19)	C3S—C4S—H4SB	108.5
C10—C9—C8	126.7 (2)	C4S^ii^—C4S—H4SB	108.5
C9—C10—C11	117.6 (2)	H4SA—C4S—H4SB	107.5
			
O7—Cu1—N1—C6	−81.46 (17)	O2—C1—C2—C3	−13.7 (3)
O5—Cu1—N1—C6	99.36 (17)	N1—C2—C3—C4	0.6 (3)
O1—Cu1—N1—C6	−176.32 (17)	C1—C2—C3—C4	178.9 (2)
O3—Cu1—N1—C6	6.16 (16)	C2—C3—C4—C5	−1.6 (3)
O7—Cu1—N1—C2	98.84 (17)	C3—C4—C5—C6	0.4 (3)
O5—Cu1—N1—C2	−80.34 (17)	C2—N1—C6—C5	−3.0 (3)
O1—Cu1—N1—C2	3.98 (16)	Cu1—N1—C6—C5	177.32 (16)
O3—Cu1—N1—C2	−173.54 (17)	C2—N1—C6—C7	174.02 (19)
O7—Cu1—N2—C9	−174.36 (18)	Cu1—N1—C6—C7	−5.7 (3)
O5—Cu1—N2—C9	3.84 (16)	C4—C5—C6—N1	1.9 (3)
O1—Cu1—N2—C9	−80.12 (17)	C4—C5—C6—C7	−174.9 (2)
O3—Cu1—N2—C9	97.72 (17)	Cu1—O3—C7—O4	−176.69 (18)
O7—Cu1—N2—C13	2.25 (16)	Cu1—O3—C7—C6	5.0 (2)
O5—Cu1—N2—C13	−179.55 (18)	N1—C6—C7—O4	−178.90 (19)
O1—Cu1—N2—C13	96.49 (17)	C5—C6—C7—O4	−1.9 (3)
O3—Cu1—N2—C13	−85.67 (17)	N1—C6—C7—O3	−0.4 (3)
N2—Cu1—O1—C1	168.99 (15)	C5—C6—C7—O3	176.5 (2)
N1—Cu1—O1—C1	−11.65 (14)	Cu1—O5—C8—O6	−178.08 (18)
O7—Cu1—O1—C1	−109.54 (15)	Cu1—O5—C8—C9	1.4 (2)
O5—Cu1—O1—C1	90.51 (15)	C13—N2—C9—C10	−1.4 (3)
O3—Cu1—O1—C1	−6.3 (2)	Cu1—N2—C9—C10	175.04 (16)
N2—Cu1—O3—C7	173.14 (15)	C13—N2—C9—C8	179.42 (19)
N1—Cu1—O3—C7	−6.11 (15)	Cu1—N2—C9—C8	−4.1 (2)
O7—Cu1—O3—C7	93.33 (15)	O6—C8—C9—N2	−178.9 (2)
O5—Cu1—O3—C7	−106.24 (15)	O5—C8—C9—N2	1.6 (3)
O1—Cu1—O3—C7	−11.5 (2)	O6—C8—C9—C10	2.0 (3)
N2—Cu1—O5—C8	−2.76 (15)	O5—C8—C9—C10	−177.6 (2)
N1—Cu1—O5—C8	−179.99 (15)	N2—C9—C10—C11	0.8 (3)
O7—Cu1—O5—C8	2.3 (3)	C8—C9—C10—C11	179.8 (2)
O1—Cu1—O5—C8	103.63 (15)	C9—C10—C11—C12	0.4 (3)
O3—Cu1—O5—C8	−102.98 (15)	C10—C11—C12—C13	−0.9 (3)
N2—Cu1—O7—C14	−4.61 (15)	C9—N2—C13—C12	0.9 (3)
N1—Cu1—O7—C14	172.61 (16)	Cu1—N2—C13—C12	−175.62 (16)
O5—Cu1—O7—C14	−9.7 (3)	C9—N2—C13—C14	176.52 (19)
O1—Cu1—O7—C14	−109.29 (15)	Cu1—N2—C13—C14	0.0 (2)
O3—Cu1—O7—C14	96.61 (15)	C11—C12—C13—N2	0.3 (3)
Cu1—O1—C1—O2	−162.50 (19)	C11—C12—C13—C14	−174.6 (2)
Cu1—O1—C1—C2	16.3 (2)	Cu1—O7—C14—O8	−175.90 (19)
C6—N1—C2—C3	1.7 (3)	Cu1—O7—C14—C13	5.8 (2)
Cu1—N1—C2—C3	−178.56 (16)	N2—C13—C14—O8	177.6 (2)
C6—N1—C2—C1	−176.78 (18)	C12—C13—C14—O8	−7.2 (4)
Cu1—N1—C2—C1	2.9 (2)	N2—C13—C14—O7	−4.0 (3)
O1—C1—C2—N1	−14.2 (3)	C12—C13—C14—O7	171.2 (2)
O2—C1—C2—N1	164.77 (19)	N1S—C1S—C2S—C2S^i^	172.2 (2)
O1—C1—C2—C3	167.4 (2)	N2S—C3S—C4S—C4S^ii^	57.8 (3)

Symmetry codes: (i) −*x*, −*y*, −*z*+1; (ii) −*x*+1, −*y*+2, −*z*.

### Hydrogen-bond geometry (Å, °)

**Table:**

*D*—H···*A*	*D*—H	H···*A*	*D*···*A*	*D*—H···*A*
O1W—H1WA···O3^iii^	0.85	1.91	2.725 (2)	160
O1W—H1WB···O6^iv^	0.85	1.89	2.720 (2)	167
O2W—H2WA···O1W^v^	0.85	1.94	2.771 (2)	165
O2W—H2WB···O2	0.85	1.98	2.828 (2)	174
O3W—H3WA···O1W^v^	0.85	2.03	2.874 (3)	171
O3W—H3WB···O2	0.85	1.97	2.779 (3)	158
N1S—H1NA···O4	0.91	1.90	2.804 (3)	171
N1S—H1NB···O7^vi^	0.83	2.55	3.112 (3)	126
N1S—H1NB···O8^vi^	0.83	2.04	2.865 (2)	176
N1S—H1NC···O2W^vii^	0.84	2.28	2.895 (3)	131
N1S—H1NC···O4^viii^	0.84	2.28	2.981 (3)	141
N2S—H2NA···O3W^vi^	0.79	1.95	2.730 (3)	166
N2S—H2NB···O5	0.86	2.31	3.149 (3)	164
N2S—H2NB···O6	0.86	2.31	3.001 (3)	138
N2S—H2NC···O2W	0.87	2.00	2.867 (3)	173
C10—H10A···O3^ix^	0.95	2.58	3.446 (3)	151
C11—H11A···O1^x^	0.95	2.46	3.139 (3)	128
C3S—H3SA···O8^xi^	0.99	2.54	3.178 (3)	122

Symmetry codes: (iii) −*x*+1, −*y*+1, −*z*+1; (iv) *x*, *y*, *z*+1; (v) −*x*+1, −*y*+2, −*z*+1;  (vi) *x*−1, *y*, *z*; (vii) *x*, *y*−1, *z*; (viii) −*x*+1, −*y*, −*z*+1; (ix) −*x*+1, −*y*+1, −*z*; (x) −*x*+2, −*y*+1, −*z*; (xi) *x*−1, *y*+1, *z*.
